# Modern surgical management of incidental gliomas

**DOI:** 10.1007/s11060-022-04045-0

**Published:** 2022-06-15

**Authors:** Anjali Pradhan, Khashayar Mozaffari, Farinaz Ghodrati, Richard G. Everson, Isaac Yang

**Affiliations:** 1 Departments of Neurosurgery, University of California, Los Angeles, Los Angeles, USA; 2 Radiation Oncology, University of California, Los Angeles, Los Angeles, USA; 3 Head and Neck Surgery, University of California, Los Angeles, Los Angeles, USA; 4 Jonsson Comprehensive Cancer Center, University of California, Los Angeles, Los Angeles, USA; 5 The Lundquist Institute, University of California, Los Angeles, Los Angeles, USA; 6 Harbor-UCLA Medical Center, University of California, Los Angeles, Los Angeles, USA; 7 David Geffen School of Medicine, Los Angeles (UCLA), Los Angeles, CA USA

**Keywords:** Glioma, Low-grade glioma, Incidental glioma, Surgical management

## Abstract

**Purpose:**

Gliomas are the most common primary tumors of the central nervous system and are categorized by the World Health Organization into either low-grade (grades 1 and 2) or high-grade (grades 3 and 4) gliomas. A subset of patients with glioma may experience no tumor-related symptoms and be incidentally diagnosed. These incidental low-grade gliomas (iLGG) maintain controversial treatment course despite scientific advancements. Here we highlight the recent advancements in classification, neuroimaging, and surgical management of these tumors.

**Methods:**

A review of the literature was performed. The authors created five subtopics of focus: histological criteria, diagnostic imaging, surgical advancements, correlation of surgical resection and survival outcomes, and clinical implications.

**Conclusions:**

Alternating studies suggest that these tumors may experience higher mutational rates than their counterparts. Significant progress in management of gliomas, regardless of the grade, has been made through modern neurosurgical treatment modalities, diagnostic neuroimaging, and a better understanding of the genetic composition of these tumors. An optimal treatment approach for patients with newly diagnosed iLGG remains ill-defined despite multiple studies arguing in favor of safe maximal resection. Our review emphasizes the not so benign nature of incidental low grade glioma and further supports the need for future studies to evaluate survival outcomes following surgical resection.

## Introduction

Gliomas are the most prevalent primary central nervous system (CNS) tumors with an age-adjusted incidence of 6/100,000 [1]. These tumors arise from atypical growth of glial cells, most commonly astrocytic and oligodendroglial cell lineages [2, 3]. As histopathologically classified in 2016 by the World Health Organization (WHO), low-grade gliomas (LGGs) consist of grades 1 and 2 gliomas, whereas more aggressive high-grade gliomas (HGGs) comprise grades 3 and 4 tumors [2, 4]. In 2021, WHO classified Gliomas into (1) Adult-type diffuse gliomas (the majority of primary brain tumors in neuro-oncology practice of adults); (2) Pediatric-type diffuse low-grade gliomas (associated with better prognoses); (3) Pediatric-type diffuse high-grade gliomas (expected to behave aggressively); and (4) Circumscribed astrocytic gliomas (“circumscribed” referring to their more solid growth pattern, as opposed to the inherently “diffuse” tumors in groups 1, 2, and 3) [5]. Gliomas classically present with symptoms such as seizures, headaches, or focal neurologic deficits [6]. Magnetic resonance imaging (MRI) is the gold-standard neuroimaging modality used for diagnosis [7]. First-line treatment for gliomas includes surgical resection alone, adjuvant radiation and/or chemotherapy [8]. Although the prognosis for HGGs has improved due to advances in diagnosis and therapy, the overall outlook remains poor [9]. Conversely, patients with LGGs have favorable survival outcomes as these tumors are slower-growing compared to HGGs [3, 10].

LGGs account for nearly 17–22% of all primary brain tumors [11]. The natural history of LGGs is defined by slow, continuous growth, and a preference for migration along white matter pathways [10]. Despite their benign nature, LGGs are at a high risk for recurrence and may progress to HGGs [3, 8]. Although almost 80% of patients with LGGs present symptomatically, a subset of them may experience no tumor-related symptoms [12]. This unique subset of lesions termed incidental low-grade gliomas (iLGGs) are defined in this study as asymptomatic low grade glioma (WHO grade 1 and 2) discovered on brain imaging for reasons not related to neuron-oncology and are incidentally diagnosed when undergoing radiographic evaluation for unrelated reasons to the underlying tumor such as trauma, annual physical examination, headache, dizziness, otolaryngological symptoms, or volunteer studies [12–15]. Conversely, LGGs manifest with neurological symptoms and deficits associated with tumors [16]. iLGGs constitute approximately 10% of LGGs [12, 13, 17, 18].

iLGGs may undergo malignant transformation, a process in which the tumor converts to a biologically aggressive HGG [19]. The incidence of this conversion is highly variable, ranging from 17 to 73%, and the reported median interval is between 2 and 10 years [7]. The literature suggests that malignant transformation occurs earlier in larger tumors, likely because the larger size is a consequence of a higher proliferative rate [20]. Additionally, malignant conversion does not always lead to a symptomatic presentation, as there has been a case report of malignant transformation to glioblastoma following 6 years of conservative management with annual MRI, in which the patient had no symptoms at the time of surgical resection [21]. Nonetheless, this process dramatically affects prognosis and has significant clinical implications.

While there has been significant improvement in understanding the pathogenesis of iLGG prognosis due to scientific advancements, the surgical management of these tumors remains a controversial topic. Recent literature highlights a shift in neurosurgeons’ opinions surrounding treatment for iLGGs from a conservative “wait and see” approach to implicating early preventative resection [22]. Early gross total resection (GTR) of iLGGs offers several benefits, however, the decision to resect iLGGs before the onset of clinical symptoms is complicated by concerns for preserving quality of life and eloquent brain structures [7, 23]. Here we highlight the current viewpoints surrounding the pathology, diagnostic imaging, and management of these tumors (Table 1).

**Table 1:** A summary of clinical outcomes from a literature survey of select recently published key studies assessing surgical management of incidental gliomas (iLGGs)

Author and Publication Year	Total patients, n	Total patients with iLGG, n	Male with iLGG, n	Age (mean ± SD), years	Common reasons for discovery of iLGG UPON initial MRI (%)	Preoperative tumor volume (cm^3^) or tumor diameter (cm)	Post-operative tumor volume (cm^3^)	Average tumor growth rate (mm/year)	Tumor resection (% of supratotal, total, or subtotal)	Median volumetric EOR (%)	Follow-up time, mo	Outcomes
Ng et al. (2020) [24]	74	74	31	34.7 ± 9.7	Headache (36.5); Dizziness (14.9); Head trauma (8.1%)	Tumor volume = 28.1 (mean)	1.3 (mean)	3.7 ± 4.5	Supratotal resection (28.4); Total resection (29.7); Subtotal resection (41.9)	95.7 (mean)	67	97.1% of patients resumed employment post-operatively. Mean time before RTW was 6.8 mo. All patients recovered from transient post-operative deficits at 3 mo. Postoperative seizure was associated with a delayed RTW. 5.4% of patients died during follow-up period. The 5-year survival rate was 100%
Wang et al. (2020) [3]	1594	80	43	41.0 median	Radiological Screening (48); Trauma (24); Dizziness (19)	NR	NR	NR	Total resection (59) and Subtotal resection (41)	NR	34.8 median	WHO Grade II patients and those with 1p/19q co-deletion tumors were more likely to be diagnosed with iLGGs. Mitochondrial aerobic respiration process was increased in iLGGs. OS and PFS were significant improved in iLGG patients. Cox regression analysis indicated iLGGs serve as an independent prognostic factor
Gogos et al. (2020) [25]	657	113	50	39.4	Headache (34.5) and Trauma (16.8)	Tumor volume = 22.5 (mean)	2.9 (mean)	3.9 (n = 43)	Total resection (57.1)	100	90	iLGGs were significant smaller than symptomatic lesions (22.5 vs 57.5 cm^3^) and iLGGs group showed greater EOR compared to sLGG. No difference in diagnosis was found between iLGG and sLGG patients regarding molecular and pathological data. OS was significantly longer for iLGG tumors. 4.4% rate of neurological deficits was seen at 6 months
Boetto et al. (2021) [10]	101	101	39	35.7 ± 11.1	Headache (33.0) and Otolaryngological symptoms (21.6)	Tumor volume = 16.4	22.9 (mean)	3.2 ± 4.4	NR	NR	35.6	Increasing tumor volume is sufficient predictor of LGG diagnosis in incidental MRI exam. Insular topography, mean initial volume greater than 4.5 cm^3^ at discovery, and adjacent sulcal effacement were predictive of further radiological progression
Zeng et al. (2021) [18]	75	75	33	38 median	Physical examination (46.7); Head trauma (18.7); Dizziness (13.3)	Tumor diameter = 2.5 (mean)	NR	2.9 ± 0.9	Total resection (83.7) and Subtotal resection (12.2)	NR	NR	Significantly more tumors were initially located adjacent to a functional area in sLGG compared to aLGG. No significant difference between sLGG and aLGG groups for total resection rate. Postoperative complication incidence was higher in sLGG group. Total resection significantly improved PFS, OS, and MPFS, but surgical timing has no effect
Ius et al. (2021)[26]	267	267	112	39.19 median	Headache (33.33), Head trauma (13.10); Otolaryngology disorders (18.35), MRI research studies (3.37), MRI follow-up (4.87), Other medical reasons (25.84%)	Tumor volume = 15 (median)	NR	NR	Total or supratotal resection (61.62%)	95%	88 (median)	The OS rate was 92.41%. The 5- and 10-year estimated OS rates were 98.09% and 93.2%, respectively. OS was significantly longer for patients with a lower preoperative tumor volume and higher EOR, regardless of the WHO-defined molecular class. OS was influenced only by the preoperative tumor volume, while TR by early surgery. A negative association was found between preoperative tumor volumes and EOR. Second surgery was performed in 26.22%. The median time between surgeries was 5.5 years. Histological evolution to high-grade glioma was detected in 22.85% of cases (16/70). Permanent mild deficits were observed in 3.08% of cases

*n* number of patients, *mo* months, *iLGG* incidental low-grade glioma, *NR* not reported, *MRI* magnetic resonance imaging, *EOR* extent of resection, *TR* tumor resectionm, *RTW* return to work, *OS* overall survival, *PFS* progression-free survival, *MPFS* malignant progression-free survival, *sLGG* symptomatic low-grade glioma, *aLGG* asymptomatic low-grade glioma

## Methods

A review of the literature was performed using Boolean operators and a combination of search terms including "surgical management” AND “incidental gliomas", "surgical management of incidental gliomas", "incidental” AND “low grade gliomas", and "incidental glioma management". Independent reviewers screened titles, abstracts, and full-text manuscripts for pertinent studies. Abstract-only texts, book chapters, animal studies, articles in languages other than English, and studies without any primary focus on incidental gliomas were excluded from this review. The authors created five subtopics of focus: histological criteria, diagnostic imaging, surgical advancements, correlation of surgical resection and survival outcomes, and clinical implications.

### Histological criteria

**Fig. 1:**
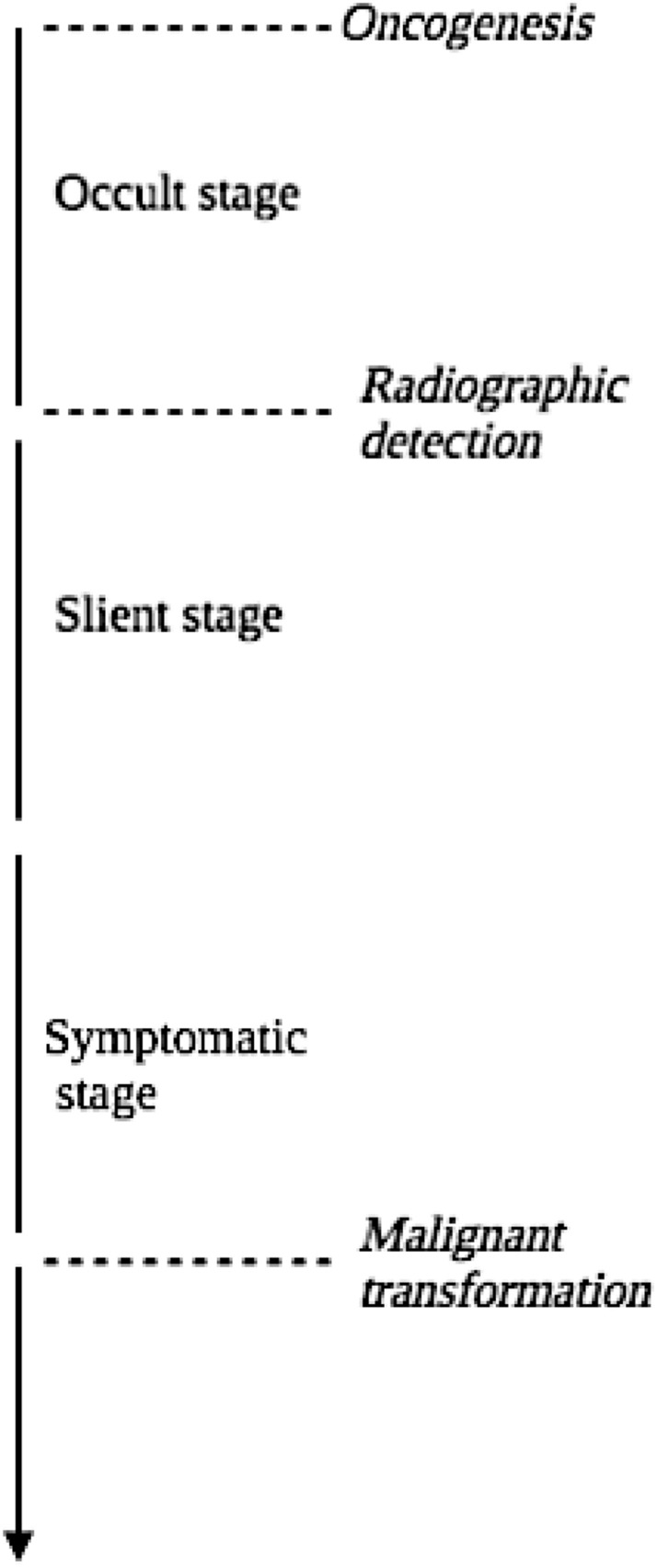
Graphic illustration of the natural history of gliomas

The natural history of glioma depicted in Fig. 1 is represented by four stages: (1) the occult stage—where the glioma elicits no symptoms and is undetectable with brain MRI, (2) the clinically silent stage—this phase encompasses asymptomatic individuals with iLGGs where the glioma is discernible on neuroimaging, but patients show no clinical symptoms, (3) the symptomatic stage—where patients commonly experience seizures, and lastly (4) the malignant transformation stage—where the LGG undergoes conversion to HGG [7, 8, 18, 19]. In 2021, WHO Classification of Tumors of the Central Nervous System significantly updated the histopathological classification of brain tumors to incorporate molecular and genetic parameters [2, 5]. This redefined grading system acknowledges several recent advances in molecular markers and genotypic features of gliomas, which are current targets for therapy [4]. There are four grades and the classification is characterized by the presence of some or all of the essential histological criteria including cytological atypia, anaplasia, mitotic figures, microvascular proliferation, and necrosis [4]. Like most LGGs, iLGGs lack many of these key histological features and mainly consist of WHO grades 1 and 2 oligodendroglioma, astrocytoma, oligoastrocytoma, and ganglioglioma [2, 16, 27]. While grade 1 lesions have no discernible histological features, grade 2 lesions are notable for cytological atypia [4]. Studies have elucidated that iLGGs have histomolecular profiles analogous to that of early-stage symptomatic LGGs, with *IDH*-mutant gliomas and 1p/19q co-deletion being predominantly associated with iLGGs [15, 18, 28].

A comprehensive understanding of the molecular and genetic features of gliomas is fundamental to guide clinical management. Several genetic markers of gliomas have been identified including: (1) mutations of isocitrate dehydrogenase 1 (IDH1) and isocitrate dehydrogenase 2 (IDH2), (2) codeletion of chromosome arms 1p and 19q, (3) alpha thalassemia retardation syndrome X-linked (ATRX) gene loss, (4) tumor suppressor protein p53 mutation, (5) promoter methylation of O6-methylguanine-DNA methyltransferase (MGMT), and (6) telomerase reverse transcriptase gene (TERT) promoter mutations [29]. IDH mutations are associated with prolonged overall survival compared to non-mutated gliomas [30]. It is hypothesized that the reason why mutations in IDH1 and IDH2, which are crucial enzymes involved in the Kreb’s cycle, are strongly correlated with improved prognosis is because of decreased NADPH production in the cell which makes tumors like LGGs more susceptible to damage from reactive oxygen species [31]. The codeletion of chromosome arms 1p and 19q are also considered to result in better prognosis and is commonly associated with IDH1/IDH2 mutations [31]. Almost all tumors with 1p/19q codeletions have mutations in TERT promoter and IDH; these tumors, known as triple-positive gliomas, may show increased benefits from treatment therapies [31]. Tumors classified with either TERT and IDH mutations or triple-positive glioma status are associated with an improved prognosis compared to lesions with only TERT mutation [32]. Further molecular classification within IDH-mutant gliomas has also shown prognostic utility as homozygous deletion of cyclin-dependent kinase inhibitor 2A (CDKN2A) was associated with poor survival in a series of 911 high-grade IDH-mutant gliomas [25]. These alterations in genetic background of LGGs compared to HGGs are important findings that have transformed our understanding of glioma progression and helped clinicians develop potential therapies for treatment. iLGGs, unlike symptomatic gliomas, utilize mitochondrial aerobic respiration, which is a process that slows the growth of tumors and may be the reason why these patients are asymptomatic and have a better prognosis [3]. In a physiological state, mitochondrial respiration consists of the preparatory reaction, the citric acid cycle, and the electron transport chain. During glycolysis which takes place in the cytoplasm outside of the mitochondria, enzymes break down glucose into two molecules of pyruvate where 2 ATPs are produced and NADH is released. These pyruvates are then converted to Acetyl CoA in the mitochondria and in the process producing more NADH. Then, in the citric acid cycle, the remaining glucose are oxidized producing 2 ATPs along with NADH and FADH2. However, most of the ATP production occurs in the electron transport chain stage of the respiration process where NADH and FADH2 give up electrons to the chain. Energy is then released and captured as the electrons move from a higher energy state to a lower energy state using a series of proteins embedded in the membranes of the mitochondria where this energy is later used to produce 32 to 34 ATPs per glucose [3, 33]. However, cancer cells exhibit mitochondrial respiration malfunction and increased glycolysis for ATP production due to aerobic glycolysis, also known as the “Warburg effect” which entails the conversion of glucose into lactic acid in an aerobic environment resulting in less sufficient ATP [3, 33, 34]. Studies have stated that glioma glycolysis or aerobic respiration is not dependent of IDH mutation status given mitochondrial aerobic respiration is not disturbed in iLGGs despite the number of IDH mutations present [3, 34]. Of note, recent investigation assessing genetic features of iLGGs compared to LGGs revealed that iLGGs comprise of a high proportion of IDH1/IDH2 mutations and 1p/19q codeletions, which suggests that iLGGs are not of an entirely different molecular and genetic subset compared to LGGs [28]. While data regarding the molecular and genetic profile of iLGGs remains sparse, recent histopathological findings support the 2016 and 2021 WHO restructuring of glioma classification and facilitate progress in understanding glioma genesis [2, 4, 5, 7, 29].

Not only do iLGGs share a similar genetic composition to LGGs, but it is also approximated that iLGGs have a radiological tumor growth rate of 4 mm/year [12]. In a series of 143 gliomas, Pallud et al. noted an inverse correlation between radiographic growth rates and survival, advocating for this parameter’s incorporation into treatment planning [35]. Additionally, iLGGs demonstrate a median Ki67 proliferative index of 5.0% [7, 15], which is comparable to that of symptomatic LGGs, indicating that early surgical resection may be beneficial to improve prognosis and survival outcomes [7, 15, 36]. Gogos et al. reported a key finding that 13% of iLGGs were observed to have IDH1/IDH2 wild-type genetic features which are associated with a worse prognosis, however, this information would not have been revealed if treatment had been delayed [36]. This finding favors early therapeutic intervention for asymptomatic iLGGs. Furthermore, the 2021 WHO Classification of Tumors of the Central Nervous System proposed a combined histological and molecular grading rather than the traditional exclusively histological tumor grading [5]. As a result, molecular parameters have now been added (which include + 7/ − 10 copy number changes in IDH-wildtype diffuse astrocytomas) as biomarkers of grading and for further estimating prognosis within multiple tumor types. This allows a glioblastoma, IDH-wildtype CNS WHO grade 4 designations even in cases that otherwise appear histologically lower grade potentially altering the definition of LGG [5].

### Diagnostic imaging

Neuroimaging plays an important role in the diagnosis of iLGGs and may provide insight to their molecular profiles [31]. In recent years, increasing access and application of MRI has led to a rise in the discovery of iLGGs [15]. Most LGGs present as isointense or hypointense on T1-weighted, and hyperintense on T2-weighted MRIs [7]. Also, LGGs, most of which are IDH-mutant, generally do not demonstrate contrast enhancement and instead have more non-enhancing solid components [31]. A notable radiographic feature among LGGs that is highly specific for IDH-mutant 1p/19q non-codeleted gliomas is the T2-FLAIR mismatch sign [37]. Genetic features not only can be seen with IDH, but also 1p/19q codeletion tumors are characterized to have indistinct margins and frequently contain calcifications [31]. While conventional MRI is the standard tool for identifying, characterizing, and measuring response to treatment for iLGGs, its ability to accurately discern radiological features can be a barrier to providing reliable information [38]. Metabolic and physiological imaging modalities are increasingly being incorporated for diagnosing, targeting, and evaluating treatment progress of LGGs. These include magnetic resonance (MR) techniques such as perfusion-weighted imaging (PWI), sodium imaging, diffusion-weighted imaging (DWI), and proton MR spectroscopy [39]. These functional techniques are capable of potentially differentiating between IDH-mutant and IDH wild-type gliomas [39]. The most promising recent advancement in proton MR spectroscopy involves 2-hydroxyglutarate (2HG) detection which is a metabolite that is characterized as a highly specific marker for IDH-mutant gliomas [10, 39]. Interestingly, there’s evidence suggesting the epileptogenic nature of 2HG as higher tissue concentrations of it have been associated with preoperative seizures in glioma patients [40]. Positron emission tomography (PET) imaging provides valuable insight about tumor metabolism and PET can be combined with radiolabeled particles such as O-(2–18F-fluoroethyl)-l-tyrosine (18F-FET), and 6-fluoro-l-DOPA(FDOPA) to guide glioma detection during biopsy or resection [41, 42]. LGGs are typically hypometabolic on PET with 18F-fluorodeoxyglucose compared to HGGs; however, PET with amino acid uptake of 18F-FET is noted to be increased in two-thirds of LGGs and is therefore utilized to distinguish LGGs from HGGs [7]. The metabolic information obtained with PET can be combined with the morphological characteristics acquired from MRI to improve histological grading and accurately perform targeted biopsies [41].

Recent imaging advancements not only serve in diagnosis of gliomas, but also can be employed during neurosurgical procedures to increase maximal extent of resection (EOR) and improve survival outcomes. Diffusion tensor imaging (DTI) uses similar principles to DWI except it is more sensitive to the diffusion of protons along white matter tracts [31]. DTI can serve as a useful aid when used in conjunction with structural MRI to plan the ideal surgical approach for maximal safe resection [7, 31]. While preoperative planning with conventional neuroimaging modalities such as MRI or PET is important, brain shift is a phenomenon that must be accounted for during surgery due to edema, gravity, or fluid changes, which results in unreliable preoperative imaging sequences and limits safe maximal EOR [24, 43]. To overcome these inherent limitations of neuronavigation, innovations including intraoperative MRI (iMRI) and intraoperative fluorescence microscopy with 5-aminolevulinic acid (5-ALA) were developed to visualize tumor tissue during resection [43]. iMRI is a frequently used technology that facilitates safe maximal EOR to preserve function in eloquent brain regions, improve prognosis, and retain quality of life [43]. Several studies have shown iMRI’s superiority to conventional MRI with respect to clinical outcomes [43]. 5-ALA provides enhanced intraoperative visualization of LGG tissue which also allows for greater EOR; however, this modality does not offer similar findings seen in HGGs which show increased levels of florescence [24]. Overall, 5-ALA fluorescence in LGGs is associated with higher grade histology and studies show that 5-ALA serves as a valuable and reliable intraoperative marker for identification of intratumoral anaplastic foci and is not vulnerable to brain shift [24]. A summary of diagnostic or biopsy/resection neuroimaging techniques for iLGGs and other grades of gliomas is provided in Table 2.

**Table 2:** A summary of common and novel diagnostic neuroimaging techniques used for diagnosis and management of incidental gliomas (iLGGs)

Neuroimaging modality	Purpose of neuroimaging modality	Mechanism and role of neuroimaging modality	Highlights and outcomes obtained with neuroimaging modality	Additional notes
Magnetic resonance imaging (MRI)-1.5 T or 3 T [7, 10, 17, 30, 35–37]	Diagnostic imaging for iLGGs	MRI is the most routine neuroimaging modality applied for diagnosis of iLGGs. MRI provides valuable anatomical and functional information about the tumor that facilitates diagnosis, resection, and monitoring of iLGGs	T-1 weighted MRI shows isointense or hypointense LGGs. T-2 weighted MRI shows hyperintense LGGs that do not typically enhance with contrast. MRI can also be used to identify prognostic features such as cyst formation or ring-like enhancement in LGGs that can help to predict survival outcomes [Deng et al.]	While 3 T offers enhanced image resolution, 1.5 T is still the most frequently used to identify and diagnose gliomas. Also, T-2-weighted Fluid-Attenuated Inversion Recovery (FLAIR) mismatch sign is a hyperintense signal on T-2 weighted MRI sequences and hypointense signal on FLAIR sequences with a hyperintense peripheral rim. This mismatch sign is a newly characterized feature that may help identify patients with IDH-mutant gliomas
Diffusion tensor imaging (DTI) [7, 10, 30, 37]	Diagnostic Imaging for iLGGs & Biopsy or Resection of iLGGs	DTI is a diffusion-weighted MRI technique that provides important information about white matter tracts in the brain through analyzing the directionality and motion of water molecules	DTI has been shown to offer useful information for surgical resection of gliomas. It serves as a complementary imaging modality to structural MRI by providing not only anatomical information, but also allowing for greater outline of functional tracts in the brain	An imaging tool for preoperative surgical resection approach planning to help neurosurgeons avoid eloquent brain regions such as motor or language areas during surgery; however, this technique is still susceptible to brain shift
Positron emission tomography (PET) imaging with amino acid *O-*(2-^18^F-fluoroethyl)-l-tyrosine (^18^F-FET) [7, 10, 37, 39]	Diagnostic imaging for iLGGs	PET is a form of metabolic imaging that can be used to aid in the diagnosis of LGGs by quantifying tumor metabolism. PET with radiotracers is capable of differentiation tumor tissue and normal brain tissue	PET imaging with use of 18F-fluorodeoxyglucose reveals that LGGs are hypometabolic in comparison to HGGs. PET imaging with 18F-FET has shown to help differentiate gliomas from other tumors. In LGGs with increased uptake of 18F-FET, studies have shown it may be suggestive of increased diagnosis accuracy for malignant progression compared to MRI	This technique could not only improve diagnosis of potential LGGs are a higher risk for malignant transformation, but also enhance histologic diagnosis obtained during biopsy or resection
Proton magnetic resonance (MR) spectroscopy (^1^H-MRSI) [7, 10, 37]	Diagnostic imaging for iLGGs	Proton MR spectroscopy is another physiologic and metabolic imaging modality that offers more information about tumor activity than conventional MRI by assessing the distribution of cellular metabolite levels	Several studies have reported ^1^H-MRSI can serve as an aid in detecting LGG tumor progression by evaluating for cellular metabolite levels that are characteristically found in LGGs, but not HGGs, such as choline (CHO) peak due to increased membrane synthesis or low N-acetylaspartate (NAA), and no presence of lactate or lipids	Specifically, studies have noted that a decreased NAA-CHO ratio potentially may be used as a technique to identify early LGG dedifferentiation and transformation to higher malignancy tumors
Intraoperative magnetic resonance imaging (iMRI) [7, 40]	Biopsy or resection of iLGGs	iMRI is a continuous imaging technique that allows for intraoperative imaging to evaluate progress of tumor resection	iMRI is well-documented in the literature as a useful technology in LGG surgery that can help safely achieve maximal EOR in an efficient and cost-effective manner	Studies have demonstrated this modality has improved EOR, decreased damage to functional brain regions, and better survival outcomes in glioma surgery
Intraoperative fluorescence-guided microscopy with 5-amino-levulinic acid (5-ALA) [7, 38, 40, 41]	Biopsy or resection of iLGGs	This novel imaging method offers enhanced intraoperative visualization of LGG tissue with the aid of 5-ALA induced fluorescence compared to traditional white-light microscopy	While 5-ALA tool has less visible fluorescence in LGGs compared to HGGs, it is still a valuable approach that has potential to be combined with newer techniques such as quantitative fluorescence, intraoperative confocal microscopy, and FLIM of PpIX to improve patient outcomes	While this technique is now routinely incorporated to visualize HGGs during resection, recent studies highlight this modality’s potential to overcome brain shift and effectively identify intratumoral regions of anaplastic foci in LGGs

*iLGG* incidental low-grade glioma, *HGG* high-grade glioma, *LGG* low-grade glioma, *MRI* magnetic resonance imaging, *MR* magnetic resonance spectroscopy, *iMRI* intraoperative MRI, *FLAIR* fluid-attenuated inversion recovery, *DTI* diffusion tensor imaging, *RTW* return to work, *IDH* isocitrate dehydrogenase, *5-ALA* 5-amino-levulinic acid, *EOR* extent of resection, ^*1*^*H-MRSI* proton MR spectroscopy, *NAA-CHO* N-acetylaspartate-choline, *PET* positron emission tomography, ^*18*^*F-FET O-*(2-^18^F-fluoroethyl)-l-tyrosine, *FLIM of PpIX* fluorescence lifetime imaging of *protoporphyrin IX*

### Surgical Advancements

Surgical resection remains the primary first-line treatment for LGGs and HGGs; however, the decision to resect iLGGs is a well-disputed debate entwined in ethical and medical concerns [16]. iLGGs are typically smaller in volume and occur in younger populations [12, 18]. The nature of iLGGs complicates the decision to either watchfully wait or surgically treat these tumors, potentially risking a decrease in quality of life due to long-term functional impact or post-operative complications [12, 18]. However, recent studies have highlighted that iLGGs are progressive tumors that share a similar fate to LGGs, and can evolve towards a higher grade of malignancy [10]. Additionally, several studies show that maximal EOR is not only associated with better overall survival, but also is safe due to the smaller size of iLGGs, which makes them less likely to be located in eloquent regions compared to symptomatic gliomas [7, 8, 12, 14–16, 36]. Several advancements have been introduced to optimize resection including enhancement of mapping of functional pathways and advanced intraoperative brain tumor visualization techniques [7, 36]. Awake intraoperative cortical stimulation mapping is an innovative modality that uses functional boundaries to achieve greater EOR in iLGG patients without inflicting treatment-related neurological deficits; this modality is also associated with decreased rates of post-operative seizures and improved neuropsychological outcomes [44]. These advancements may allow for avoidance of not only motor, language, or cognitive disabilities, but also social and professional disabilities in patients’ diagnosis [45]. While epilepsy is a serious complication from early prophylactic surgery in iLGG patients, it is observed in less than 10% of patients during long-term follow-up, and should not prevent patients from undergoing early resection [46]. Studies that combine DTI and conventional MRI to delineate glioma’s adjacent cortical tracts are associated with lower likelihood of neurological deficits or damage to motor pathways [7, 47]. Lastly, fluorescence-guided 5-ALA resections for LGGs are being optimized to improve fluorescence visualization through use of intraoperative confocal microscopy and other advances to improve EOR and determine tumor histopathology [24, 43]. These advancements may facilitate the neurosurgery community’s inclination to resect these tumors earlier, instead of watchful waiting.

### Correlation of surgical resection and survival outcomes

Although optimal management of iLGGs is still a dilemma, there has been a growing consensus amongst neurosurgeons that these tumors are not as indolent as originally characterized to be, which has sparked new evaluation of survival outcomes in iLGG patients who undergo early surgical resection [48]. Even small volume iLGGs are not benign lesions, but instead are tumors that carry a risk of progression to a higher grade and possible anaplastic transformation resulting in death [49]. Therefore, iLGG’s risk of malignant progression should encourage clinicians to treat these tumors in a manner similar to symptomatic LGGs [36, 49]. Studies have shown that early maximal EOR improves overall survival (OS) in symptomatic LGGs through delaying risk of malignant transformation [7, 36, 44, 48]. It is also possible for malignant transformation to occur even if the patient remains asymptomatic [21]. Numerous studies have reported that an early prophylactic surgery approach results in a greater EOR and prolonged survival for iLGGs compared to symptomatic LGGs [10, 14, 16, 36, 48]. iLGGs may be more amenable to GTR because they are less likely to be located in eloquent brain regions and therefore are associated with improved OS [7]. While certain genetic parameters are associated with a better prognosis of iLGGs, early preventative surgery is also a crucial component [8, 46]. One study reported a 20 cm^3^ volume increase over an average of 28 months which supports early resection when iLGG tumor volumes tend to be lower and accordingly allow for greater rates of EOR [16]. Zeng et al. reported an intriguing comparison of surgical timing between two cohorts of iLGG patients: (1) those who underwent surgical resection prior to symptom onset and (2) those who delayed surgical treatment until symptoms arose [17]. This study found that surgical timing was not significantly associated with OS, progression-free survival (PFS), and malignant PFS (MPFS) rates [17]. However, total resection was a significant factor that showed positive correlation to OS, PFS, and MPFS; therefore, surgical timing should be utilized to assist neurosurgeons with achieving maximal EOR [17]. Additionally, in a series of patients, Jakola et al. and Ius et al. noted better overall survival in patients with LGG and iLGG undergoing early resection compared to those managed conservatively through biopsy and “wait and see” approach [23, 26]. The aforementioned studies provide compelling data advocating for early surgical resection of iLGGs [14, 29, 36, 48]. Similarly, the new 2021 WHO classification of molecular subtypes among the EOR classes, and the proposed combined histological and molecular grading alters the definition of LGGs, therefore impacting the management strategy (surgical intervention versus surveillance) and survival outcomes [5, 26].

Although some groups have shared concern about sacrificing cognitive function in young patients with iLGGs for early resection, recent data shows neuropsychological results were not impacted by awake surgery [44]. A novel study assessed the return to work rate in iLGG patients who underwent awake resection with intraoperative mapping and demonstrated that while postoperative seizures were associated to a delayed return to work, 97.1% of patients were still able to resume their professional activities, suggesting that early surgery to prevent malignant progression is capable of producing favorable outcomes [45]. Additionally, Zeng et al. noted a higher rate of postoperative complications and postoperative seizures in patients with symptomatic LGGs compared to asymptomatic ones [17]. This data further supports early surgical resection as delaying surgery until the onset of symptoms may increase the risk of complications. In combination with the likely shared natural history of iLGGs with symptomatic LGGs, these survival outcome findings collectively support early preventative surgical resection of iLGGs.

Regarding individualizing treatment options for patients diagnosed with iLGG, there is a lack of consensus. Some studies oppose early resection and favor surveillance with serial clinical examination and MR imaging because iLGGs harbor IDH1 mutation leading to a delayed disease progression and malignant transformation [12, 18, 50]. Whereas, other studies recommend early surgical intervention as a primary consideration given iLGGs are a precursor to symptomatic LGGs and iLGGs’ non-quiescent nature even in asymptomatic patients [8, 10, 12, 14–17, 23, 36, 44, 45]. However, despite serval studies reporting in favor of early resection over the past decades, surgical resection followed by radiation therapy has been the mainstay of treatment for high-risk LGG with subtotal resection, early radiation being associated with longer progress-free survival [51–53]. Furthermore, a large phase III trial has reported an overall survival of 7.8 years in patients treated with radiation alone, compared with 13.3 years in patients treated with both radiation and chemotherapy [54]. However, surgical treatment for concurrent lesions or treatment of early radiation at diagnosis versus at the time of recurrence is yet to be elucidated.

### Clinical implications

An optimal treatment approach for patients with newly diagnosed iLGG remains ill-defined. In iLGG patients, the decision to resect early is a medical, ethical, and socioeconomic challenge which must be carefully weighed to assess the immediate and delayed consequences of choosing a conservative versus surgical approach [50]. Unnecessary intervention may cause a disruption in quality of life and foster pre- and post-operative anxiety; however, active surveillance may also be seen as an ethical dilemma [50]. Future studies should comprehensively evaluate additional parameters such as employment abilities, as well as social, legal, and cultural issues that are inherent to this decision [45]. Some authors have proposed implementing a radiological screening policy for healthy individuals ages 20–40 [10]. Ultimately, a screening policy would also require a personalized treatment paradigm to ensure reliable patient-centered care that avoids ensuing lifelong disabilities in young patients who prior to diagnosis, were largely unaffected [50].

Treatment of iLGG is controversial due to their asymptomatic nature and lack of associated histologic confirmation leaving the physician to counsel a patient based solely on MRI [55]. Shah et al. suggest a conservative protocol of active surveillance which includes repeated physical examinations and surveillance MRI every 4 months [55]. Other studies have shown that the continuous growth of iLGGs (> 2 mm/year) can provide sufficient justification on MRI; therefore, physicians could utilize this growth, the onset clinical symptoms or a positive 18-fluorodeoxyglucose positron emission tomography scan to administer treatments [15, 55, 56]. Furthermore, several studies have implied the safe and practical aspect of a conservative protocol of active surveillance prior to actively treating these lesions [15, 55–63].

## Conclusions

Significant progress in management of gliomas, regardless of the grade, has been made through modern neurosurgical treatment modalities, diagnostic neuroimaging, and a better understanding of the genetic composition of these tumors. However, the clinical protocol for surgical management of iLGGs remains controversial. Although more is known about the natural history of iLGGs, additional information is necessary to thoroughly assess the impact of early surgical resection on prognosis. Our review emphasizes the not benign nature of iLGGs and further supports the need for future studies to evaluate survival outcomes following surgical resection.

## Data Availability

Not applicable.
